# Choosing sweeteners wisely—nutrigenetic study on childhood obesity

**DOI:** 10.1186/s12986-025-01015-x

**Published:** 2025-10-06

**Authors:** Daniel Wang Qiu, Chia-Min Kuo, Shih-Yuan Hsu, Emily Chia-Yu Su, San-Yuan Wang, Jia-Woei Hou, Meng-Che Tsai, Chen Yang, Yang-Ching Chen

**Affiliations:** 1 College of Medicine, Taipei Medical University, Taipei, Taiwan; 2 Department of Family Medicine, School of Medicine, College of Medicine, Taipei Medical University, Taipei, Taiwan; 3 Graduate Institute of Biomedical Informatics, Taipei Medical University, Taipei, Taiwan; 4 Master Program in Clinical Genomics and Proteomics, Department of Pharmaceutical Sciences, Taipei Medical University, Taipei, Taiwan; 5 Department of Pediatrics, Cathay General Hospital, Taipei, Taiwan; 6 College of Medicine, Fu-Jen Catholic University, New Taipei City, Taiwan; 7 Department of Pediatrics, College of Medicine, National Cheng Kung University Hospital, National Cheng Kung University, Tainan, Taiwan; 8 Department of Pediatrics, Taipei Medical University Hospital, Taipei Medical University, Taipei, Taiwan; 9 Ihmed Children Healthcare Center, Taipei, Taiwan; 10 Department of Family Medicine, Wan Fang Hospital, Taipei Medical University, Taipei Medical University, Taipei 116, Taiwan, No. 111 Section 3, Xing-long Road, Wenshan District, Taipei, 11696 Taiwan R.O.C.; 11 School of Nutrition and Health Sciences, College of Nutrition, Taipei Medical University, Taipei, Taiwan; 12 Graduate Institute of Metabolism and Obesity Sciences, Taipei Medical University, Taipei, Taiwan; 13 Nutrition Research Center, Taipei Medical University Hospital, Taipei, Taiwan

**Keywords:** Childhood obesity, Gene–nutrient interaction, Dietary preference, Cohort study

## Abstract

**Background:**

This study investigated the association of specific sweet-taste and obesity-related genes with sweetener consumption patterns among children and the interaction between these genetic factors and sweetener intake on the risk of childhood obesity. By leveraging data from the Taiwanese Pubertal Longitudinal Study (TPLS), the current study minimized the influence of environmental confounders commonly encountered in adult studies, offering a more precise understanding of these relationships in pediatric and adolescent populations.

**Methods:**

Participants in the TPLS underwent genetic sampling, anthropometric measurements, puberty stage assessments, dietary recall, and measurements of relevant lifestyle variables. Nonnutritive sweetener (NNS) intake was assessed using the validated Nonnutritive Sweetener Food Frequency Questionnaire (NNS-FFQ). The statistical analysis employs logistic regression to investigate the correlations between genotypes and sweetener consumption, while accounting for potential confounders such as parental education and household income. Simultaneously, the study examines gene-sweetener interactions to assess the association between specific alleles and particular sweetener consumption patterns.

**Results:**

Higher consumption of specific artificial sweeteners—acesulfame potassium, sucralose, and steviol—was associated with lower body mass index (BMI) Z-scores and reduced body fat percentage. The interaction analyses indicated a significantly positive association of the interaction between sucralose consumption and sweet-taste genes on the waist–hip ratio. Genetic analysis revealed significant associations between obesity-related genes (e.g., ADCY9 and TFAP2B) and sweet-taste receptor genes (e.g., TAS1R2 and TAS1R3) with sweetener consumption, which may influence susceptibility to obesity. Notably, rs7498665 was significantly associated with BMI Z-scores, underscoring its role in obesity predisposition.

**Conclusions:**

These findings highlight the genetic underpinnings of sweetener consumption and its interactive effects with genetic variants on childhood obesity risk, providing valuable insights for promoting public health and developing personalized nutrition strategies. Future research involving larger samples and consideration of genetic and environmental factors is required to develop personalized nutrition strategies aimed at effectively combating childhood obesity.

**Supplementary Information:**

The online version contains supplementary material available at 10.1186/s12986-025-01015-x.

## Background

Economic and technological advancements have considerably increased access to a diverse range of foods, contributing to a shift toward Westernized dietary patterns that are typically high in fat, salt, and sugar. Moreover, stress from modern professional and academic demands often leads to overeating, further fueling the obesity epidemic. Consequently, obesity has become an increasingly prominent public health concern requiring urgent attention [1]. Childhood obesity, in particular, has reached epidemic proportions worldwide. In 2016, more than 340 million children and adolescents aged 5–19 years were classified as overweight or obese [2]. Evidence shows that childhood obesity significantly increases the risk of adult obesity, with children with obesity being approximately 5 times more likely to experience obesity in adulthood than their nonobese peers [3]. Approximately 55% of children with obesity continue to experience obesity in adolescence, and approximately 80% of adolescents with obesity experience it in adulthood [3]. These statistics highlight the long-term health implications of early-life obesity.

Since sweet taste perception influences individual food preferences and dietary habits, genetic variations in sweet taste perception may contribute to differences in the intake of essential nutrients and micronutrients [4]. However, studies on the relationship between genetic variations in sweet taste perception and sweetener consumption are limited in scope and often limited by small sample sizes. Furthermore, the majority of research conducted on this subject has concentrated on adult populations [5–8]. Factors such as individual lifestyle choices, food availability, emotional associations with eating, cumulative dietary education, cultural exposure, and prior sweetener consumption tend to vary widely [9, 10]. These elements are shaped over time by personal, social, and occupational experiences, making it difficult to isolate the effects of genetic predispositions in adult cohorts. Meanwhile, previous studies have suggested that genetic influences on sweet preference may be overridden by cultural factors and life experiences in adults [11]. In contrast, by studying on children in this research, the dietary behaviors are typically more homogeneous and largely shaped by caregivers and family eating patterns. As a result, the influence of these environmental confounders is comparatively reduced in childhood populations, allowing for a clearer examination of how genetic factors influence sweet taste perception and related dietary intake.

The food processing industry has increasingly incorporated nonnutritive sweeteners (NNSs), in addition to traditional sweeteners, as additives in products such as chewing gum, beverages, and other packaged goods. In packaged foods, NNSs are commonly added to foods to enhance sweetness and improve the palatability of foods [12]. These NSSs offer the advantage of providing sweetness comparable to that of traditional sweeteners while maintaining lower or even zero calorie content. Therefore, NNSs have become a popular substitute for traditional sweeteners, particularly for those seeking weight control. However, studies have reported counterintuitive effects of NNSs on metabolic outcomes and associated comorbidities, such as obesity [13], impaired glucose homeostasis [14], cardiovascular disease [15–17], and alterations in gut microbiota [18]. Nevertheless, research on the interaction between gene variations and NNS intake on the risk of childhood obesity is limited.

Considering the notable correlation of sugar consumption on childhood obesity, this study investigated the relationship between specific sweet-taste genes and obesity-related genes and sweetener consumption patterns in Asian children and adolescents. In addition, the study examined the interaction between these genetic factors and NNS consumption as well as their combined effect on the risk of childhood obesity. This study analyzed data from the Taiwanese Pubertal Longitudinal Study (TPLS) cohort to minimize the influence of environmental confounders, which are commonly encountered in adult studies, to offer a more precise understanding of these relationships in pediatric and adolescent populations.

## Methods

### Study population and data collection

The TPLS is a multidisciplinary, longitudinal project involving data collected from female adolescents aged 6–16 years and male adolescents aged 9–17 years who were recruited from several hospitals (Taipei Medical University Hospital, Cathay General Hospital, Taipei Municipal Wanfang Hospital, and National Cheng Kung University Hospital [NCKUH]). The participants in the TPLS undergo routine anthropometric measurements and annual evaluations, including 24-h dietary recall, with a particular focus on dietary habits and the consumption of sweet foods and sweeteners. Additionally, lifestyle factors such as smoking habits, sleep patterns, physical activity levels, and stress levels are assessed as covariates for questionnaire calibration.

To obtain supplementary information, the parents or guardians of each participant were asked to complete a baseline characteristics questionnaire with their children. This questionnaire was used to gather information regarding the participant’s birth date, sex, postnatal information such as birth weight and breastfeeding status, and family socioeconomic status, including family income and parental education level. Environmental factors such as exposure to tobacco smoke at home and the use of incense at home were also documented. Furthermore, the Chinese version of the International Physical Activity Questionnaire was employed to assess the average duration of physical activity among the children [19]. Trained registered dietitians conducted 2 separate 24-h dietary recall assessments following the initial recruitment to collect detailed dietary intake data. Energy and nutrient intakes were estimated using the Nutritionist Edition of COFIT Pro, Version 1.0.0, a software package that employs the Taiwanese food composition table as its nutrient database. The accuracy of this software was previously validated in a study published by our team [20].

## Nonnutritive sweetener food frequency questionnaire

Because no specialized questionnaire for assessing sweetener intake is currently available for Asian populations, our research team developed the Nonnutritive Sweetener Food Frequency Questionnaire (NNS-FFQ) [21] to assess the consumption of commercially available Taiwanese food products containing one or more nonnutritive sweeteners (NNSs). A comprehensive market survey was conducted to identify NNS-containing food products commonly consumed in Taiwan, including beverages, chips and corn snacks, cookies, candies, nutritional supplements, frozen foods, seasoned seafood and meats, instant noodles, and dehydrated or flavored vegetables and fruits. These products were systematically collected and subjected to both qualitative and quantitative analyses to determine their NNS content. The NNS-FFQ was designed to capture intake data on the five most commonly used sweeteners in Taiwan—acesulfame potassium, aspartame, sucralose, glycyrrhizin, steviol glycosides, and sorbitol. It also includes items assessing the intake of added sugars. In the present study, individual NNS exposure levels were calculated using the dietary data obtained from the NNS-FFQ. Based on the results of the market survey and chemical analyses, we established a comprehensive food composition database specific to NNS-containing products. This database was subsequently integrated with dietary intake data from participants in the Taiwanese Pubertal Longitudinal Study (TPLS) to estimate individual NNS consumption.

## Weight index measurement and nutrient intake assessment

The participants’ weights and heights were measured using an electronic scale while they were barefoot and wearing lightweight clothing and after they had fast in the leadup to the assessment. Body height and weight were measured with high precision, with height recorded to the nearest 0.1 cm and weight to the nearest 0.1 kg. Body mass index (BMI) was subsequently calculated as weight in kilograms divided by height in meters squared (kg/m^2^). The classification of overweight and obesity was determined using age- and sex-specific cutoff points corresponding to the 85th and 95th percentiles, respectively, as outlined in the Growth Charts for Taiwanese Children [22]. BMI values were converted into BMI Z-scores by using the World Health Organization Growth Standards [23]. Waist and hip circumferences were measured using a flexible tape to the nearest 1 mm. Body fat percentage was assessed using a portable bioimpedance analysis electronic scale (TT-BC 418, TANITA Corporation, Tokyo, Japan).

## Candidate single nucleotide polymorphism genotyping, confounding factor assessment, and statistical analysis

The genes previously identified as associated with obesity include *TAS1R2*, *TAS1R3*, *GLUT2*, *GNAT*, *FTO*, and *FGF21* [5, 8, 24]. In our study of obesity-related genes, we selected 18 single nucleotide polymorphisms (SNPs): rs2531995, rs9356744, rs11604680, rs1421085, rs7206790, rs9939609, rs16858082, rs8053360, rs6567160, rs3817334, rs1555543, rs574367, rs4788102, rs7498665, rs12597579, rs4715210, rs12463617, and rs6548238. Previous studies [25–34] have indicated that these genes adjacent to these SNPs are associated with obesity and related body metrics in various Asian populations, including Chinese, Indonesian, and Korean. Additionally, we included 9 SNPs associated with sweet taste perception: rs838133, rs838145, rs10242727, rs1107657, rs6467192, rs6467217, rs12033832, rs12137730, and rs7534618. The obesity-related genes near these SNPs include *ADCY9* [35], *CDKAL1* [34], *CELF1* [36], *FTO* [37], *GNPDA2* [38], *IRX3* [39], *MC4R* [40], *MTCH2* [40], *PTBP2* [40], *SEC16B* [40], *SH2B1* [41], *GP2* [42], *TFAP2B* [43], and *TMEM18* [34]. Regarding the sweet-taste genes, *FGF21* [44], *GNAT3* [45], and *TAS1R2* [7] are relevant to our study. These SNPs and nearby genes are listed in Supplementary Tables S1 and S2.

The participants of the TPLS were instructed to refrain from eating or drinking for a minimum of 1 h prior to providing a sample. In providing a sample, they used a brush to scrape the inside of their mouths 10 times to collect buccal cells. The collection brushes were subsequently stored at −80 °C before being transferred for DNA purification [46]. DNA extraction was performed using the Gentra Puregene Buccal Cell Kit 140 in accordance with the specified protocol of the kit. The extracted DNA was subsequently diluted to a concentration of 2.5 ng/µL for polymerase chain reaction analysis. Genotyping validation was performed using Sequenom iPLEX matrix-assisted laser desorption/ionization-time of flight mass spectrometry at the National Center for Genome Medicine. This process was facilitated through the National Genotyping Center of Academia Sinica (http://lims.ngc.sinica.edu.tw/service/) platform [47].

Polygenic risk scores (PRSs) were calculated to assess the correlations between various SNPs. This score was derived by summing the relevant alleles associated with obesity-related and sweet-taste genes, with each allele weighted by its effect size, as indicated by its beta (*β*) coefficient. An additive model was employed to calculate this weighted sum, with the sum reflecting the cumulative genetic architecture [48].

The statistical models used in this study included a priori confounders identified using the TPLS data and NNS-FFQ. The following a priori confounders were adjusted in all models: parental education level, family income level, breastfeeding status, birth weight, gestational age, and exposure to secondhand smoke. Mean differences in continuous variables between the sweetener intake group and the control group were compared using Student’s *t* test. The primary outcomes and key exposure related to sweeteners intake and genetic variation were categorized. For instance, participants were classified into groups based on “nonconsumers, low consumers, and high consumers” of particular sweeteners and investigated differences in overweight vs. nonoverweight status. Consequently, logistic regression analysis was employed to investigate the relationship between genetic variations and sweetener intake. Interaction terms were included in the logistic regression model to investigate the effect of the interaction between nutritional intake and genotypes on sweetener consumption. Furthermore, both full and reduced models were used to examine whether the interaction between sweetener intake and genotypes exhibited a synergistic or an antagonistic effect. A *P* value of < 0.05 was considered significant.

By reviewing previous studies and selecting candidate SNPs based on established quality control criteria, the verification process involved several steps: ensuring sex concordance, optimizing call rates for both markers and individuals (typically exceeding 98%), excluding SNPs with a low minor allele frequency (MAF < 1%), assessing Hardy–Weinberg equilibrium (HWE) using a p-value threshold of 1 × 10⁻⁶, removing individuals with heterozygosity deviations exceeding ± 3 standard deviations (SD) from the mean, and excluding related individuals with a pi-hat value greater than 0.2, which indicates second-degree relatives or closer.

## Results

### Demographic characteristics

**Fig. 1:**
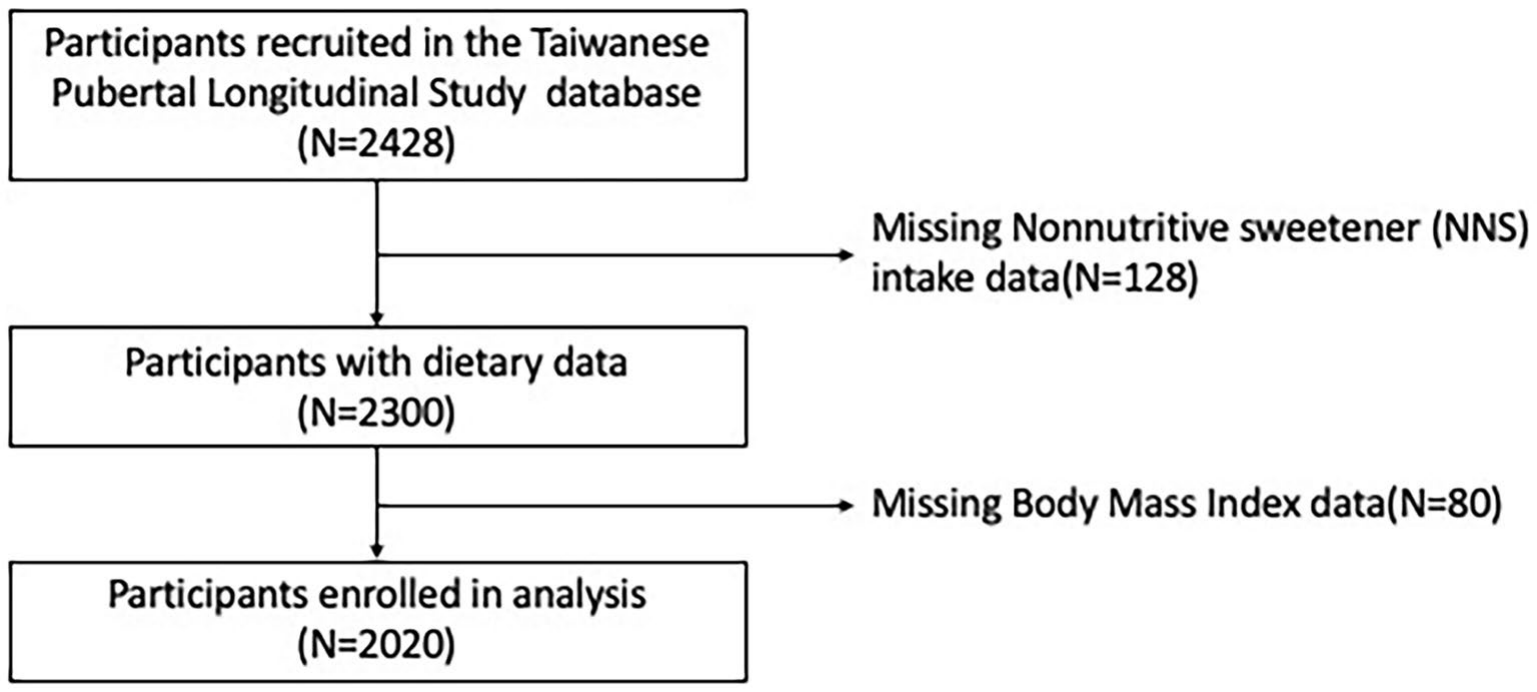
Flowchart of data collection and participant selection from the Taiwanese Pubertal Longitudinal Study (TPLS)

Figure 1 illustrates the data collection and participant selection process from the Taiwanese Pubertal Longitudinal Study database. Initially, 2,428 participants were recruited, but 128 were excluded due to missing NNS intake data, leaving 2,300 with available dietary data. After excluding 80 more participants with missing BMI data, the study population consisted of 1361 girls and 661 boys, with a mean age of 10.39 years. Table 1 presents the demographic data, including parental education level, family income level, breastfeeding status, exposure to secondhand smoke, daily metabolic equivalent (METD), and BMI. Participants were categorized on the basis of their consumption of artificial sweeteners, namely acesulfame potassium, aspartame, and sucralose, as nonconsumers, low consumers, and high consumers, with the median intake used as the cutoff point.

**Table 1:** Baseline characteristics of participants

	Participants		Category of artificial sweetener intake						*P*
			Nonconsumers		Low consumers		High consumers		
	Mean/*N*	SD/%	Mean/*N*	SD/%	Mean/*N*	SD/%	Mean/*N*	SD/%	
N	**2022**		**768**		**627**		**627**		
Age	10.39	2.08	10.18	2.10	10.49	2.05	10.53	2.08	0.27
Sex									0.44
Female	1361	67.31%	552	0.70	406	0.60	403	0.60	
Male	661	32.69%	216	0.30	221	0.40	224	0.40	
Parental edu									0.33
Low	136	7.97%	57	8.55%	36	6.67%	43	8.60%	
Medium	987	57.82%	377	56.52%	317	58.70%	293	58.60%	
High	584	34.21%	233	34.93%	187	34.63%	164	32.80%	
Income									0.01
Low	194	11.43%	84	12.65%	68	12.64%	42	8.48%	
Medium	664	39.13%	265	39.91%	215	39.96%	184	37.17%	
High	839	49.44%	315	47.44%	255	47.40%	269	54.34%	
Breastfeeding									0.42
No	316	18.46%	113	16.89%	106	19.56%	97	19.36%	
Yes	1396	81.54%	556	83.11%	436	80.44%	404	80.64%	
Secondhand smoke									0.62
No	1231	75.38%	484	76.10%	388	74.76%	359	75.10%	
Yes	402	24.62%	152	23.90%	131	25.24%	119	24.90%	
METD	5.35	6.38	4.94	5.85	5.19	5.80	6.10	7.55	0.88
Body mass index	18.12	3.65	18.28	3.73	18.43	3.66	17.62	3.50	< 0.01
Z-score	0.25	1.42	0.36	1.37	0.36	1.43	< 0.01	1.44	< 0.01
Nonobese	1647	81.45%	647	84.24%	517	82.46%	483	77.03%	< 0.01
Overweight	521	25.77%	208	27.08%	182	29.03%	131	20.89%	0.02
Obese	261	12.91%	109	14.19%	87	13.88%	65	10.37%	0.12

Parental edu = the highest level education attained by either parent, categorized as low: senior high school or below, medium: college, and high: graduate school or higherIncome = monthly household income, classified as low: <New Taiwanese Dollars (NT$)70 000, medium: between NT$70 000 and NT$100 000, and high: >NT$100 000METD = daily metabolic equivalentCategories of artificial sweetener intake = total consumption of artificial sweeteners, including acesulfame potassium, aspartame, and sucraloseChildhood obesity categories: Based on age- and sex-specific BMI percentiles, as defined by the World Health Organization: nonobese: below the 85th percentile, overweight: between the 85th and 95th percentile, and obese: above the 95th percentile

No significant differences in age were observed between nonconsumers, low consumers, and high consumers of artificial sweeteners. Similarly, no significant difference in sex distribution was noted among the different sweetener intake categories. Regarding parental education level, breastfeeding status, exposure to secondhand smoke, and METD, no significant differences were observed between the artificial sweetener intake categories. However, significant differences were observed for family income level and BMI across the categories. Higher levels of artificial sweetener intake were statistically associated with lower BMI and higher family income levels, though these relationships do not imply causation due to the observational nature of the study. Additionally, the prevalence of overweight exhibited significant differences between the different categories.

## Association between artificial sweetener consumption and body composition

Table 2 presents the types of sweeteners analyzed in this study, namely acesulfame potassium, aspartame, sucralose, glycyrrhizin, steviol glycosides, sorbitol, added sugar, and Total NNSs. Sweetener consumption levels were categorized as “no consumption” (no), “low consumption” (low), and “high consumption” (high), with the median value serving as the cutoff point. The table presents the *β* values, standard errors, and *P* values for each sweetener in relation to body composition indices, including BMI Z-score, the waist-hip ratio, and fat percentage.

**Table 2:** Association between nonnutritive sweetener consumption and body composition indices

Sweetener	Categ	BMI Z-score			Fat percentage			Waist–hip ratio		
		β	SE	P*	β	SE	P*	β	SE	P*
AceK	No			< 0.01			< 0.01			0.56
	Low	−0.05	0.10	0.61	−0.66	0.72	0.36	−2.01	1.69	0.23
	High	−0.28	0.10	< 0.01	−2.32	0.72	< 0.01	−0.46	1.67	0.78
Asp	No			0.04			0.05			0.03
	Low	0.16	0.09	0.06	0.85	0.65	0.19	−0.98	1.53	0.52
	High	−0.25	0.09	0.01	−1.67	0.67	0.01	3.96	1.55	0.01
Suc	No			< 0.01			< 0.01			0.63
	Low	−0.07	0.09	0.40	−0.36	0.64	0.57	4.22	1.53	0.01
	High	−0.37	0.09	< 0.01	−2.64	0.64	< 0.01	−0.05	1.49	0.97
Gly	No			< 0.01			0.02			0.96
	Low	0.00	0.09	0.97	0.02	0.67	0.97	0.98	1.54	0.52
	High	−0.32	0.09	< 0.01	−1.78	0.66	0.01	−0.37	1.58	0.81
Ste	No			< 0.01			< 0.01			0.99
	Low	−0.19	0.12	0.09	−1.70	0.87	0.05	0.49	2.01	0.81
	High	−0.32	0.11	< 0.01	−2.43	0.84	< 0.01	−0.15	1.92	0.94
Sor	No			< 0.01			< 0.01			0.52
	Low	0.04	0.08	0.66	0.17	0.62	0.79	0.62	1.46	0.67
	High	−0.29	0.08	< 0.01	−1.89	0.62	< 0.01	−1.09	1.49	0.46
Added sugar	No			0.06			0.45			0.53
	Low	0.07	0.09	0.44	0.39	0.68	0.57	0.39	1.57	0.80
	High	−0.14	0.09	0.11	−0.40	0.66	0.55	0.97	1.58	0.54
Total NNS	No			< 0.01			< 0.01			0.49
	Low	0.06	0.08	0.48	0.35	0.62	0.57	2.25	1.50	0.13
	High	−0.31	0.08	< 0.01	−2.32	0.62	< 0.01	0.90	1.47	0.54

ZBMI = body mass index Z-score; SE = Standard error; AceK = Acesulfame potassium; Asp = Aspartame; Suc = Sucralose; Gly = Glycyrrhizin; Ste = Steviol glycosides; Sor = Sorbitol; Categ = Category of consumption amount, with the median value as the cutoff*P for trend refers to the statistical significance when the trend is considered across no, low, and high consumption categories for their impact on body composition

In terms of NNS consumption, the consumption of acesulfame potassium, aspartame, sucralose, glycyrrhizin, steviol, sorbitol, and total NNSs was found to be associated with lower BMI Z-score and fat percentage. A significant dose-response association was observed, with a higher intake of acesulfame potassium, sucralose, and steviol associated with a lower BMI Z-score. The *β* values of BMI Z-score were − 0.05 and − 0.28 for low and high consumption of acesulfame potassium, respectively. Similar results were noted for sucralose (*β* = −0.07 and − 0.37) and steviol (*β* = −0.19 and − 0.32). Additionally, a significant dose-response association was noted for lower fat percentage. Acesulfame potassium, sucralose, and steviol were associated with lower fat percentage, with the *β* values for low and high consumption respectively being − 0.66 and − 0.32 for acesulfame potassium, −0.36 and − 0.64 for sucralose, −1.70 and − 2.43 for steviol.

## Effect of the interaction between NNSs and sweet-taste genes/obesity-related genes on body composition

Tables 3 and 4 present the interaction effects between various sweeteners and genes on body composition indices, including BMI Z-score, fat percentage, and the waist–hip ratio. The analysis revealed several significant gene–sweetener interactions influencing key health metrics. When the interaction between NNSs and obesity-related genes were analyzed, significant effects were observed. Notably, the interaction between total NNSs and obesity-related genes exerted a positive effect on the waist–hip ratio (*P* for multiplicative interaction = 0.05 and *P* for trend = 0.03).

**Table 3:** Effects of interaction between nonnutritive sweetener consumption and obesity genes on different body composition indices

SWT	Interaction		BMI Z-score				Fat percentage				Waist–hip ratio			
	NNS	OB gene	P*	β(95%Cl)	*P*	*P* for trend	P*	β(95%Cl)	*P*	*P* for trend	P*	β(95%Cl)	*P*	*P* for trend
AceK	Low	Low	0.81	Ref	-	0.07	0.92	Ref	-	0.40	0.31	Ref	-	0.85
	Low	High		0.37(0.13, 0.61)	< 0.01			2.55(0.92, 4.18)	< 0.01			3.11(−1.95,8.17)	0.23	
	High	Low		0.04(−0.23, 0.31)	0.79			0.70(−1.24, 2.64)	0.48			1.08(−4.07, 6.23)	0.68	
	High	High		0.35(0.04, 0.66)	0.03			1.68(−0.53, 3.89)	0.14			0.61(−6.41, 7.63)	0.86	
Asp	Low	Low	0.16	Ref	-	0.14	0.32	Ref	-	0.06	0.28	Ref	-	0.04
	Low	High		0.46(0.21, 0.71)	< 0.01			3.05(1.31, 4.79)	< 0.01			*-*0.32(−5.85, 5.21)	0.91	
	High	Low		0.11(−0.14, 0.36)	0.37			0.91(−0.87, 2.69)	0.32			2.35(−2.37, 7.07)	0.33	
	High	High		0.29(−0.0, 0.58)	0.06			2.50(0.38, 4.62)	0.02			6.81(0.58, 13.04)	0.03	
Suc	Low	Low	0.12	Ref	-	0.92	0.24	Ref	-	0.87	0.61	Ref	-	0.02
	Low	High		0.49 (0.24, 0.74)	< 0.01			3.24 (1.4, 5.08)	< 0.01			0.72 (−5.02, 6.46)	0.81	
	High	Low		0.04 (−0.21, 0.29)	0.76			*-*0.46 (−2.22, 1.3)	0.61			3.92 (−0.76, 8.6)	0.10	
	High	High		0.14(−0.13, 0.41)	0.31			1.14(−0.82, 3.1)	0.26			6.85(0.72, 12.98)	0.03	
Gly	Low	Low	0.69	Ref	-	0.73	0.52	Ref	-	0.39	0.98	Ref	-	0.20
	Low	High		0.38 (0.14, 0.62)	< 0.01			2.81 (1.1, 4.52)	< 0.01			1.51 (−3.92, 6.94)	0.59	
	High	Low		*-*0.11 (−0.36, 0.14)	0.40			−0.22 (−2.06, 1.62)	0.82			2.08 (−2.82, 6.98)	0.41	
	High	High		0.19 (−0.1, 0.48)	0.22			1.64 (−0.48, 3.76)	0.13			3.72 (−2.55, 9.99)	0.25	
Ste	Low	Low	0.99	Ref	-	0.22	0.78	Ref	-	0.36	0.68	Ref	-	0.15
	Low	High		0.35(0.13, 0.57)	< 0.01			2.40(0.89, 3.91)	< 0.01			1.27(−3.41, 5.95)	0.60	
	High	Low		*-*0.11(−0.44, 0.22)	0.51			*-*1.35(−3.68, 0.98)	0.26			2.42(−3.5, 8.34)	0.42	
	High	High		0.24(−0.15, 0.63)	0.23			1.58(−1.16, 4.32)	0.26			5.96(−2.61, 14.53)	0.17	
Sor	Low	Low	0.31	Ref	-	0.8	0.24	Ref	-	0.93	0.82	Ref	-	0.62
	Low	High		0.45(0.18, 0.72)	< 0.01			3.27(1.33, 5.21)	< 0.01			2.18(−3.94, 8.3)	0.49	
	High	Low		*-*0.09(−0.34, 0.16)	0.46			*-*0.56(−2.3, 1.18)	0.53			0.66(−4.02, 5.34)	0.78	
	High	High		0.15(−0.12, 0.42)	0.29			1.08(−0.88, 3.04)	0.28			1.84(−4.08, 7.76)	0.54	
Added sugar	Low	Low	0.26	Ref	-	0.06	0.45	Ref	-	0.06	0.27	Ref	-	0.39
	Low	High		0.18(−0.17, 0.53)	0.31			1.69(−0.8, 4.18)	0.18			5.15(−2.16, 12.46)	0.17	
	High	Low		0.07(−0.2, 0.34)	0.60			*-*0.28(−2.18, 1.62)	0.77			3.06(−2.17, 8.29)	0.25	
	High	High		0.35(0.06, 0.64)	0.02			2.56(0.52, 4.6)	0.01			3.17(−3.14, 9.48)	0.33	
Total NNS	Low	Low	0.27	Ref	-	0.20	0.35	Ref	-	0.29	0.68	Ref	-	0.03
	Low	High		0.48(0.19, 0.77)	< 0.01			3.25(1.15, 5.35)	< 0.01			0.86(−5.51, 7.23)	0.79	
	High	Low		0.05(−0.2, 0.3)	0.67			0.11(−1.65, 1.87)	0.90			3.55(−1.19, 8.29)	0.14	
	High	High		0.31(0.04, 0.58)	0.03			2.03(0.11, 3.95)	0.04			6.22(0.2, 12.24)	0.04	

Definitions: SWT = Sweeteners; OB gene = Obesity genes; AceK = Acesulfame potassium; Asp = Aspartame; Suc = Sucralose; Gly = Glycyrrhizin; Ste = Steviol glycosides; Sor = Sorbitol*P: *P* for the multiplicative interaction of the interaction between genes and sweeteners on body composition indices

**Table 4:** Effects of interaction between nonnutritive sweetener consumption and sweet-taste genes on different body composition indices

SWT	Interaction		BMI Z-score				Fat percentage				Waist–hip ratio			
	NNS	SWT gene	P*	β(95%Cl)	*P*	*P* for trend	P*	β(95%Cl)	*P*	*P* for trend	P*	β(95%Cl)	*P*	*P* for trend
AceK	Low	Low	0.74	Ref	-	0.73	0.96	Ref	-	0.50	0.90	Ref	-	0.38
	Low	High		0.02 (−0.22, 0.26)	0.88			0.25 (−1.42, 1.92)	0.77			4.48 (−0.4, 9.36)	0.07	
	High	Low		−0.01 (−0.28, 0.26)	0.93			−0.69 (−2.57, 1.19)	0.47			−0.49 (−5.8, 4.82)	0.86	
	High	High		0.08 (−0.23, 0.39)	0.62			−0.51 (−2.92, 1.9)	0.68			4.56 (−2.32, 11.44)	0.20	
Asp	Low	Low	0.44	Ref	-	0.95	0.93	Ref	-	0.67	0.17	Ref	-	0.01
	Low	High		−0.02 (−0.27, 0.23)	0.85			0.30 (−1.5, 2.1)	0.74			2.29 (−3.04, 7.62)	0.40	
	High	Low		−0.08 (−0.33, 0.17)	0.55			0.26 (−1.5, 2.02)	0.77			1.85 (−3.07, 6.77)	0.46	
	High	High		0.05 (−0.24, 0.34)	0.72			0.43 (−1.67, 2.53)	0.68			10.05 (3.93, 16.17)	< 0.01	
Suc	Low	Low	0.61	Ref	-	0.20	0.89	Ref	-	0.22	0.03	Ref	-	< 0.01
	Low	High		−0.01 (−0.26, 0.24)	0.95			0.37 (−1.53, 2.27)	0.70			0.29 (−5.34, 5.92)	0.92	
	High	Low		−0.20 (−0.45, 0.05)	0.12			−1.01 (−2.73, 0.71)	0.25			1.30 (−3.58, 6.18)	0.60	
	High	High		−0.11 (−0.38, 0.16)	0.44			−0.85 (−2.87, 1.17)	0.41			10.93 (5.07, 16.79)	< 0.01	
Gly	Low	Low	0.13	Ref	-	0.28	0.47	Ref	-	0.57	0.83	Ref	-	0.06
	Low	High		−0.07 (−0.31, 0.17)	0.56			−0.09 (−1.85, 1.67)	0.92			4.26 (−0.93, 9.45)	0.11	
	High	Low		−0.28 (−0.55, −0.01)	0.04			−1.06 (−2.9, 0.78)	0.26			1.79 (−3.25, 6.83)	0.49	
	High	High		−0.04 (−0.33, 0.25)	0.78			−0.06 (−2.14, 2.02)	0.95			6.99 (0.82, 13.16)	0.03	
Ste	Low	Low	0.72	Ref	-	0.63	0.79	Ref	-	0.40	0.97	Ref	-	0.03
	Low	High		0.02 (−0.2, 0.24)	0.86			0.33 (−1.22, 1.88)	0.68			4.65 (0.1, 9.2)	0.05	
	High	Low		−0.14 (−0.47, 0.19)	0.40			−0.93 (−3.18, 1.32)	0.42			3.14 (−2.98, 9.26)	0.32	
	High	High		−0.03 (−0.42, 0.36)	0.89			−1.12 (−4.06, 1.82)	0.46			8.02 (−0.27, 16.31)	0.06	
Sor	Low	Low	0.59	Ref	-	0.14	0.42	Ref	-	0.14	0.52	Ref	-	0.27
	Low	High		−0.01 (−0.28, 0.26)	0.94			0.85 (−1.15, 2.85)	0.40			6.08 (0.08, 12.08)	0.05	
	High	Low		−0.23 (−0.48, 0.02)	0.08			−0.85 (−2.56, 0.86)	0.33			1.06 (−3.8, 5.92)	0.67	
	High	High		−0.14 (−0.41, 0.13)	0.32			−1.15 (−3.13, 0.83)	0.26			4.42 (−1.21, 10.05)	0.12	
Added sugar	Low	Low	0.57	Ref	-	0.69	0.04	Ref	-	0.71	0.11	Ref	-	0.14
	Low	High		−0.05 (−0.4, 0.3)	0.79			−1.95 (−4.48, 0.58)	0.13			−0.65 (−8.24, 6.94)	0.87	
	High	Low		−0.03 (−0.3, 0.24)	0.85			−1.03 (−2.89, 0.83)	0.28			−1.46 (−6.67, 3.75)	0.58	
	High	High		0.05 (−0.22, 0.32)	0.74			0.22 (−1.86, 2.3)	0.84			5.34 (−0.48, 11.16)	0.07	
Total NNS	Low	Low	0.42	Ref	-	0.93	0.61	Ref	-	0.74	0.05	Ref	-	0.01
	Low	High		−0.05 (−0.34, 0.24)	0.72			−0.16 (−2.3, 1.98)	0.88			−0.26 (−6.59, 6.07)	0.93	
	High	Low		−0.10 (−0.35, 0.15)	0.45			−0.66 (−2.38, 1.06)	0.45			1.08 (−3.84, 6.0)	0.67	
	High	High		0.01 (−0.26, 0.28)	0.96			−0.07 (−2.05, 1.91)	0.94			9.29 (3.57, 15.01)	< 0.01	

Definitions: SWT = Sweeteners; SWT gene = Sweet-taste genes; AceK = Acesulfame potassium; Asp = Aspartame; Suc = Sucralose; Gly = Glycyrrhizin; Ste = Steviol glycosides; Sor = Sorbitol

*P: P for the multiplicative effect of the interaction between genes and sweeteners on body composition indices

An analysis of the interactions between sweetener consumption and sweet-taste genes also revealed notable effects on body composition. The interaction between sucralose intake and sweet-taste genes had a positive effect on the waist–hip ratio (*P* for multiplicative interaction = 0.03 and *P* for trend = < 0.01). Additionally, the interaction between added sugar intake and sweet-taste genes positively influenced fat percentage (*P* for multiplicative interaction = 0.04). Finally, the interaction between total NNSs and sweet-taste genes exhibited a positive correlation with the waist–hip ratio (*P* for multiplicative interaction = 0.05 and *P* for trend = 0.01).

### Association between NNS consumption and genetic variants

Both positive and negative significant associations between SNPs and NNS consumption were observed (Supplementary Tables 1 and 2). Supplementary Table 3 presents data on the associations between phenotypes and genotypes, with a specific focus on the PRSs for BMI Z-scores in relation to obesity-related and sweet-taste genes. In Supplementary Table 3, each row corresponds to a particular SNP, with the table detailing information such as the nearby gene, allele, minor allele frequency (MAF), *β* coefficient for BMI Z-score, and *P* value. For obesity-related phenotypes, SNP rs7498665 in *SH2B1* exhibited a significant positive association with BMI Z-score (*β* = 0.20, *P* = 0.02). Another SNP, rs8053360, in *IRX3*, exhibited a marginal association with BMI Z-score (*β* = 0.16, *P* = 0.07), although the association was not significant at the threshold of *P* < 0.05. Regarding sweetener consumption, the PRS for BMI Z-score was associated with several SNPs, but none of these associations reached significance at *P* < 0.05 after adjustment for multiple testing. Notable among these associations is the suggestive positive association between the SNP rs838133 in *FGF21* and sweetener consumption (*β* = 1.15, *P* = 0.04), indicating a potential genetic predisposition toward higher NNS intake.

A significant PRS for BMI Z-score, with a *β* coefficient of 21.40 and a highly significant *P* value of 4.41E – 07, was observed in the obesity gene group, indicating a strong influence on BMI Z-score and a substantial role in obesity predisposition. These findings underscore the complex genetic architecture underlying both obesity and sweetener consumption, highlighting the importance of further investigation into the molecular mechanisms and potential therapeutic targets for these phenotypes.

## Discussion

This study explored the interaction between NNSs and various obesity-related body metrics, with a specific focus on childhood obesity. By examining the combined influence of NNS consumption and genetic predispositions, this study provides crucial insights into how genetic variations in sweet-taste and obesity-related genes modulate the effect of sweetener consumption on obesity risk. The findings reveal a significant relationship between dietary sweeteners and genetic factors, offering a deeper understanding of personal risk factors for obesity. These findings underscore the importance of considering both genetic background and dietary habits in managing pediatric obesity, and they indicate potential avenues for developing more targeted interventions and personalized nutrition strategies.

Our study identified a dose-response association between the intake of acesulfame potassium, sucralose, and steviol and lower BMI Z-scores, as well as a similar association with reduced fat percentage. These findings may indicate a potential association between artificial sweetener consumption and pediatric weight status, which is consistent with previous research suggesting that artificial sweeteners can reduce caloric intake without compromising sweetness satisfaction [49, 50]. Moreover, significant differences were observed in the prevalence of overweight and obesity across sweetener intake categories, further highlighting the potential association between artificial sweetener consumption and weight status.

Notably, in this study, the interaction between sucralose and sweet-taste genes as well as the relationship between Total NNS and genetic variations, had a positive impact on the waist–hip ratio and body fat percentage. This suggests that genetic variations influence body composition and fat accumulation, potentially modulating the effects of sweetener intake [51, 52]. Several mechanisms may explain these contradictory results. First, genetic variations may affect individual metabolic pathways for sweeteners, thereby altering their impact on weight and fat accumulation [53]. Second, genetic variations may influence taste perception, resulting in individual differences in sensitivity and preferences for sweeteners. This could affect eating behaviors and total energy intake [9], contributing to varying effects on body weight and fat distribution. These findings highlight the complex and potentially harmful interactions between sweetener consumption, genetic predispositions related to obesity, and sweet taste perception, indicating that careful consideration is required when dietary sweeteners are selected.

This study further explored the correlation between genetic variants and sweetener consumption, highlighting the influence of genetic factors on sweetener preferences. Regarding obesity-related genes, rs2531995 (*ADCY9*) was positively correlated with the consumption of aspartame, steviol, sucralose, and added sugar, whereas rs4715210 (*TFAP2B*) and rs574367 were negatively correlated with acesulfame potassium and added sugar intake. In terms of sweet-taste genes, glycyrrhizin consumption was positively associated with several variants, including rs1107657, rs6467192, rs6467217, rs12033832, and rs7534618l. However, rs838145 and rs10242727 were negatively correlated with glycyrrhizin and sorbitol consumption. Additionally, rs7498665, an obesity-related variant, was identified as positively influencing body composition metrics, such as BMI Z-scores, although this effect was not significantly associated with sweet perception genes. Studies have indicated that variations in sweet-taste genes influence children’s sweet perception, with both sensitivity to sugars and intake levels [54–56] being associated with genetic variations. For instance, variations in *TAS1R2* have been linked to differences in sensitivity and the intake of high-sugar foods during childhood [54], a relationship observed in both children and adults [6]. Furthermore, this study noted a significant PRS based on selected obesity-related SNPs for BMI Z-score, suggesting a strong influence of obesity-related genes on obesity predisposition. This finding indicates that consideration individual genetic variants alone cannot provide a comprehensive risk assessment; rather, the combined effect of multiple genetic variants must be considered in predicting obesity risk.

NNSs are increasingly being used as additives in various food products. However, studies have indicated that NNSs can trigger metabolic responses similar to those induced by caloric sweeteners. For instance, animal studies have demonstrated that NNS consumption can disrupt the gut microbiota composition, potentially affecting metabolic health by influencing nutrient absorption and energy regulation [57]. Additionally, human studies have reported associations between NNS intake and increased risks of obesity and metabolic syndrome. These associations are hypothesized to be mediated by mechanisms such as impaired appetite regulation and glucose metabolism [58, 59].

This study has several notable advantages. First, by focusing on children rather than adults, it minimizes the confounding effects of these environmental factors. In childhood populations, lifestyle habits and food choices are typically more uniform and heavily influenced by caregivers, reducing the variability introduced by individual life experiences or long-term dietary education. This allows for a clearer investigation of how genetic factors influence sweet taste perception and related dietary intake, thereby elucidating the gene–diet interactions that contribute to obesity. Furthermore, the study specifically examined the effects of various sweeteners on obesity, offering insights into how interactions between sweeteners and genetic variants impact obesity-related traits.

Second, this study’s investigation of the interaction between sweet-taste genes and artificial sweetener consumption in relation to childhood obesity rates provides valuable insights for clinical research. By integrating data on sweetener consumption with genetic information, this study makes a substantial contribution to the field of preventive medicine. Moreover, the findings of this research can assist with the development of personalized dietary plans tailored to the profiles of children and adolescents. Implementing such plans early in life can lead to dietary improvements specific to certain genetic predispositions, which can mitigate the adverse effects of obesity in contemporary society. In conclusion, this study highlights the potential role of artificial sweeteners in pediatric weight management strategies.

The study has several limitations that should be acknowledged. The primary limitation is the relatively modest sample size, which may compromise the generalizability of the study findings. Although the inclusion of 1361 girls and 661 boys provides a sufficient foundation for conducting PRS analyses on obesity-related genes associated with BMI Z-scores, a larger sample size would enhance statistical power and yield more precise estimates of the associations between sweet-taste genes, sweetener consumption, and obesity. Another limitation of this study is the lack of direct quality control. While efforts were made to ensure data reliability by referencing and adopting quality control procedures from previous research, no direct quality control measures were implemented specifically for this study. This reliance on pre-existing methodologies may introduce unrecognized biases or inconsistencies that were not addressed in the current research design. In addition, we didn’t conduct formal testing for population stratification. Although all participants were of Han Chinese ancestry from a localized region in Taiwan, suggesting a relatively genetically homogeneous cohort, we did not conduct principal component analysis (PCA) to empirically assess potential substructure. Consequently, subtle residual stratification cannot be entirely ruled out. While the targeted candidate SNP approach and reliance on previously validated variants in East Asian populations likely mitigated this risk, future studies involving larger and more diverse samples should incorporate genetic principal components to account for any potential population structure. Furthermore, the cross-sectional design of the study precludes causal inference, as temporal relationships between genetic markers, sweetener consumption, and obesity outcomes cannot be established. Finally, we did not obtain significant results for the PRS related to sweet-taste genes and their association with BMI Z-score, with the exception of those for SNP rs838133 in *FGF21*, which exhibited a suggestive positive association with sweetener preference. Several factors other than sample size might have contributed to this lack of significant findings. One such factor is genetic heterogeneity, which can dilute association signals, rendering the detection of significant associations challenging. Additionally, environmental factors, such as diet, physical activity levels, and socioeconomic status, play a critical role in obesity development and must be considered in analyses [60]. Obtaining a larger sample size would enable a more accurate exploration of potential interactions between genetic and environmental factors. These limitations highlight the need for further research involving larger and more diverse cohorts to validate the current findings and explore the underlying mechanisms.

## Conclusion

This study investigated the daily dietary intake of various NNSs and added sugars among participants in the TPLS. The findings of this study contribute to the limited body research on child health in Asia by integrating data on sweeteners with genetic information. Although further research is required with larger sample sizes and consideration of genetic heterogeneity and environmental influence to validate this study’s results, the current findings enhance the understanding of the role of artificial sweeteners in pediatric weight management. Furthermore, the findings underscore the influence of genetic factors on sweetener preferences and obesity. Overall, this study offers valuable insights that can assist in developing targeted dietary interventions and personalizing nutrition plans aimed at combating obesity in children.

## Supplementary Information

Supplementary Material 1.

## Data Availability

No datasets were generated or analysed during the current study.

## Abbreviations

NNS: Nonnutritive sweetener
TPLS: Taiwanese Pubertal Longitudinal Study
GWAS: Genome-wide association studies
NNS-FFQ: Nonnutritive Sweetener Food Frequency Questionnaire
AceK: Acesulfame potassium
Asp: Aspartame
Suc: Sucralose
Gly: Glycyrrhizin
Ste: Steviol glycosides
Sor: Sorbitol
BMI: Body mass index
METD: Daily metabolic equivalent
SNP: Single nucleotide polymorphism
BMI Z-score: Body mass index Z-score
