# Supplementary material for: Choosing sweeteners wisely—nutrigenetic study on childhood obesity
Source: Nutr Metab (Lond). 2025 Oct 6;22:114. doi: 10.1186/s12986-025-01015-x (PMC12502141; doi:10.1186/s12986-025-01015-x)
Supplement: Supplementary file 1 — Supplementary Material 1. [file 12986_2025_1015_MOESM1_ESM.docx]

**Supplementary Table 1. Correlation between obesity-related genes and sweetener consumption**

| **RS no.** | **Nearby gene** | ***β* (*P*)** | | | | | | | |
| --- | --- | --- | --- | --- | --- | --- | --- | --- | --- |
|  |  | **Association with sweetener** | | | | | | | |
|  |  | **ACE-K** | **Aspartame** | **Glycyrrhizin** | **Steviol** | **Sucralose** | **Sorbitol** | **Added sugar** | **Total NNS** |
| rs2531995 | *ADCY9* | 0.44 (0.06) | 0.81 (0.02) | <0.01 (0.65) | 0.08 (0.02) | 0.77 (0.03) | 50 (0.19) | 1.59 (0.03) | 53.7 (0.16) |
| rs9356744 | *CDKAL1* | −0.19 (0.67) | −0.52 (0.43) | −0.01 (0.73) | −0.05 (0.51) | −0.63 (0.36) | −20.52 (0.78) | 1.01 (0.45) | −20.91 (0.78) |
| rs11604680 | *CELF1* | 0.04 (0.90) | −0.25 (0.58) | <0.01 (0.98) | −0.02 (0.66) | 0.66 (0.18) | 8.98 (0.86) | 1.03 (0.32) | 10.44 (0.84) |
| rs1421085 | *FTO* | 0.07 (0.84) | −0.25 (0.59) | <0.01 (0.99) | −0.02 (0.67) | 0.67 (0.18) | 9.96 (0.85) | 1.19 (0.25) | 11.6 (0.83) |
| rs7206790 | *FTO* | −0.17 (0.73) | −0.56 (0.43) | <0.01 (0.86) | −0.06 (0.47) | −0.35 (0.62) | −56.75 (0.47) | −0.06 (0.97) | −57.94 (0.46) |
| rs9939609 | *FTO* | 0.04 (0.72) | −0.05 (0.78) | <0.01 (0.62) | 0.01 (0.85) | 0.11 (0.65) | 8.69 (0.79) | −0.38 (0.50) | 8.42 (0.79) |
| rs16858082 | *GNPDA2* | −0.02 (0.96) | 0.22 (0.61) | <0.01 (0.83) | 0.09 (0.06) | 0.45 (0.32) | 19.86 (0.67) | 0.35 (0.71) | 20.95 (0.66) |
| rs8053360 | *IRX3* | −0.19 (0.47) | −0.42 (0.27) | <0.01 (0.83) | −0.06 (0.15) | −0.22 (0.59) | 20.54 (0.62) | −1.55 (0.07) | 18.09 (0.67) |
| rs6567160 | *MC4R* | −0.39 (0.53) | −0.39 (0.69) | 0.09 (0.05) | −0.06 (0.71) | −0.91 (0.50) | −5.64 (0.97) | −3.12 (0.29) | −10.42 (0.95) |
| rs3817334 | *MTCH2* | 0.05 (0.85) | 0.10 (0.79) | <0.01 (0.83) | 0.03 (0.45) | 0.58 (0.14) | 61.99 (0.13) | 0.51 (0.53) | 63.25 (0.13) |
| rs1555543 | *PTBP2* | 0.53 (0.23) | 0.73 (0.25) | −0.02 (0.36) | 0.06 (0.38) | −0.10 (0.88) | 2.42 (0.97) | −0.30 (0.83) | 3.32 (0.96) |
| rs574367 | *SEC16B* | −0.12 (0.63) | −0.15 (0.65) | <0.01 (0.99) | −0.01 (0.73) | −0.30 (0.41) | −60.86 (0.11) | −1.61 (0.03) | −63.05 (0.10) |
| rs4788102 | *SH2B1* | 0.32 (0.19) | 0.50 (0.16) | <0.01 (0.60) | 0.05 (0.23) | 0.06 (0.88) | −0.12 (0.99) | −1.23 (0.10) | −0.42 (0.99) |
| rs7498665 | *SH2B1* | −0.28 (0.33) | −0.76 (0.06) | −0.01 (0.59) | −0.08 (0.07) | −0.02 (0.96) | −77.43 (0.09) | −1.30 (0.14) | −79.88 (0.08) |
| rs12597579 | *GP2* | 0.20 (0.40) | 0.17 (0.61) | <0.01 (0.89) | 0.02 (0.57) | 0.70 (0.05) | 25.93 (0.49) | −0.86 (0.25) | 26.17 (0.49) |
| rs4715210 | *TFAP2B* | −0.65 (0.02) | −0.70 (0.09) | −0.01 (0.36) | −0.08 (0.09) | −0.83 (0.06) | −51.67 (0.26) | −1.96 (0.03) | −55.9 (0.23) |
| rs12463617 | *TMEM18* | 0.08 (0.71) | −0.02 (0.96) | −0.01 (0.56) | −0.03 (0.61) | 0.16 (0.72) | −23.31 (0.68) | −0.42 (0.68) | −23.54 (0.68) |
| rs6548238 | *TMEM18* | −0.33 (0.48) | −0.37 (0.58) | −0.02 (0.24) | −0.04 (0.47) | −0.13 (0.84) | −31.96 (0.55) | 1.16 (0.45) | −31.7 (0.56) |

* Regarding the relationship between obesity-related genes and sweetener consumption, several genetic variants were significantly positively correlated with specific sweetener consumption. First, the variant rs2531995, located in ADCY9, exhibited significant associations with Asp (β = 0.81, P = 0.02), Ste (β = 0.08, P = 0.02), Suc (β = 0.77, P = 0.03), and added sugar consumption (β = 1.59, P = 0.03). Second, the variant rs4715210 in TFAP2B exhibited a significant negative correlation with AceK (β = −0.65, P = 0.02) and added sugar consumption (β = −1.96, P = 0.03). Moreover, the variant rs574367 was negatively correlated with added sugar consumption (β = −1.61, P = 0.03).

**Supplementary Table 2. Correlations between sweet taste genes and sweetener consumption**

| **RS no.** | **Nearby**  **gene** | ***β* (*P*)** | | | | | | | |
| --- | --- | --- | --- | --- | --- | --- | --- | --- | --- |
|  |  | **Association with sweetener** | | | | | | | |
|  |  | **ACE-K** | **Aspartame** | **Glycyrrhizin** | **Steviol** | **Sucralose** | **Sorbitol** | **Added sugar** | **Total NNS** |
| rs838133 | *FGF21* | 2.26 (0.16) | 3.24 (0.16) | −0.01 (0.91) | 0.04 (0.88) | 3.35 (0.18) | 64.85 (0.80) | 1.12 (0.83) | 72.61 (0.78) |
| rs838145 | *FGF21* | −0.08 (0.62) | −0.22 (0.31) | −0.02 (0.01) | −0.02 (0.46) | −0.08 (0.73) | −55.3 (0.03) | 0.01 (0.99) | −55.71 (0.03) |
| rs10242727 | *GNAT3* | −0.09 (0.55) | −0.26 (0.25) | −0.02 (0.01) | −0.02 (0.43) | −0.10 (0.68) | −55.78 (0.03) | 0.07 (0.89) | −56.2 (0.03) |
| rs1107657 | *GNAT3* | 0.22 (0.31) | 0.29 (0.34) | 0.02 (0.02) | 0.02 (0.57) | 0.47 (0.16) | −16.57 (0.63) | −0.86 (0.20) | −16.4 (0.64) |
| rs6467192 | *GNAT3* | −0.23 (0.35) | <0.01 (0.99) | 0.04 (<0.01) | −0.06 (0.18) | −0.50 (0.20) | −8.04 (0.84) | 0.78 (0.32) | −8.01 (0.84) |
| rs6467217 | *GNAT3* | 0.27 (0.31) | 0.29 (0.34) | 0.02 (0.02) | 0.02 (0.57) | 0.47 (0.16) | −16.57 (0.63) | −0.86 (0.21) | −16.4 (0.64) |
| rs12033832 | *TAS1R2* | 0.21 (0.31) | 0.28 (0.36) | 0.02 (0.02) | 0.02 (0.63) | 0.48 (0.15) | −17.49 (0.61) | −0.89 (0.19) | −17.36 (0.62) |
| rs12137730 | *TAS1R2* | −0.06 (0.73) | −0.17 (0.51) | −0.01 (0.18) | −0.05 (0.09) | −0.45 (0.10) | 4.82 (0.87) | 0.45 (0.42) | 4.53 (0.87) |
| rs7534618 | *TAS1R2* | 0.14 (0.49) | 0.15 (0.62) | 0.02 (0.03) | 0.01 (0.78) | 0.38 (0.24) | −19.58 (0.57) | −0.77 (0.24) | −19.65 (0.57) |

*****Regarding the relationship between the sweet-taste genes and sweetener consumption, the variant rs838145 in FGF21 exhibited a significant negative correlation with Gly (β = −0.02, P = 0.01) and Sor (β = −55.3, P = 0.03) consumption, suggesting a potential genetic influence on this sweetener preference. Similarly, the variant rs10242727 in GNAT3 exhibited a significant negative correlation with Gly (β = −0.02, P = 0.01) and Sor (β = −55.78, P = 0.03) consumption. Conversely, significant positive correlations were noted between Gly consumption and several genetic variants, including rs1107657 (β = 0.02, P = 0.02), rs6467192 (β = 0.04, P < 0.01), rs6467217 (β = 0.02, P = 0.02), rs12033832 (β = 0.02, P = 0.02), and rs7534618 (β = 0.02, P = 0.03), suggesting a potential genetic influence on sweetener preferences.

**Supplementary Table 3. Correlations between obesity and sweet-taste genes in relation to ZBMI**

| **Phenotype** | **RS no.** | **Nearby gene** | **Allele** | **MAF** | ***β*** | ***P*** | **PRS to ZBMI** | |
| --- | --- | --- | --- | --- | --- | --- | --- | --- |
|  |  |  |  |  |  |  | ***β*** | ***P*** |
| OB | rs2531995 | *ADCY9* | T/C | 0.21 | −0.11 | 0.14 | 21.40 | 4.41E − 07 |
| OB | rs9356744 | *CDKAL1* | C/A | 0.03 | 0.12 | 0.37 |  |  |
| OB | rs11604680 | *CELF1* | T/C | 0.07 | 0.01 | 0.90 |  |  |
| OB | rs1421085 | *FTO* | T/C | 0.07 | 0.02 | 0.88 |  |  |
| OB | rs7206790 | *FTO* | C/A | 0.02 | 0.23 | 0.11 |  |  |
| OB | rs9939609 | *FTO* | C/A | 0.23 | 0.02 | 0.75 |  |  |
| OB | rs16858082 | *GNPDA2* | T/C | 0.09 | 0.07 | 0.47 |  |  |
| OB | rs8053360 | *IRX3* | T/C | 0.13 | 0.16 | 0.07 |  |  |
| OB | rs6567160 | *MC4R* | A/G | <0.01 | 0.17 | 0.63 |  |  |
| OB | rs3817334 | *MTCH2* | T/C | 0.13 | 0.03 | 0.70 |  |  |
| OB | rs1555543 | *PTBP2* | T/C | 0.04 | −0.15 | 0.29 |  |  |
| OB | rs574367 | *SEC16B* | T/C | 0.25 | 0.10 | 0.20 |  |  |
| OB | rs4788102 | *SH2B1* | T/C | 0.30 | −0.01 | 0.90 |  |  |
| OB | rs7498665 | *SH2B1* | T/C | 0.11 | 0.21 | 0.02 |  |  |
| OB | rs12597579 | *GP2* | T/C | 0.23 | −0.01 | 0.85 |  |  |
| OB | rs4715210 | *TFAP2B* | T/C | 0.10 | −0.11 | 0.22 |  |  |
| OB | rs12463617 | *TMEM18* | C/A | 0.04 | −0.04 | 0.72 |  |  |
| OB | rs6548238 | *TMEM18* | C/A | 0.02 | 0.20 | 0.20 |  |  |
|  |  |  |  |  |  |  | ***β*** | ***P*** |
| SWT | rs838133 | *FGF21* | T/C | <0.01 | 1.15 | 0.04 | 1.50 | 0.59 |
| SWT | rs838145 | *FGF21* | C/T | 0.45 | <0.01 | 0.95 |  |  |
| SWT | rs10242727 | *GNAT3* | T/C | 0.45 | −0.01 | 0.83 |  |  |
| SWT | rs1107657 | *GNAT3* | C/T | 0.15 | −0.05 | 0.47 |  |  |
| SWT | rs6467192 | *GNAT3* | C/T | 0.11 | 0.05 | 0.57 |  |  |
| SWT | rs6467217 | *GNAT3* | C/T | 0.15 | −0.05 | 0.47 |  |  |
| SWT | rs12033832 | *TAS1R2* | T/C | 0.15 | −0.05 | 0.49 |  |  |
| SWT | rs12137730 | *TAS1R2* | T/C | 0.26 | 0.03 | 0.60 |  |  |
| SWT | rs7534618 | *TAS1R2* | C/T | 0.15 | −0.05 | 0.47 |  |  |

*Definitions: OB = obesity genes; SWT = sweet-taste genes; MAF = minor allele frequency; PRS = polygenic risk score

*Genetic correlations revealed associations between specific SNPs and sweetener consumption preferences, indicating a genetic influence on taste perception. Polygenic risk score (PRS) analysis further highlighted significant associations between obesity-related SNPs and BMI Z-scores, indicating genetic predispositions to obesity.
